# Presumed oxaliplatin-related Guillain–Barré syndrome: A case report and review of chemotherapy-related Guillain–Barré syndrome

**DOI:** 10.1097/MD.0000000000051121

**Published:** 2026-10-16

**Authors:** Sikander Chohan, Muhammad Wasif Saif

**Affiliations:** a Department of Internal Medicine, Wayne State University School of Medicine, Detroit, MI; b Department of Oncology, Barbara Ann Karmanos Cancer Center, Detroit, MI.

**Keywords:** chemotherapy, cholangiocarcinoma, Guillain–Barré syndrome, neuropathy, oxaliplatin, pharmacology, toxicity

## Abstract

**Rationale:**

Oxaliplatin, a platinum-based chemotherapeutic widely used in colorectal, hepatobiliary, and pancreatic malignancies, commonly causes a symmetric, cold-induced sensory neuropathy. Rarely, it has been linked to immune-mediated Guillain-Barré Syndrome (GBS), which can be misattributed to drug-induced sensory neuropathy and delay diagnosis. Distinguishing the two is critical because GBS can progress to respiratory failure and death.

**Patient concerns:**

A 77-year-old woman with concurrent metastatic cholangiocarcinoma and colorectal carcinoma developed acute, symmetric, ascending motor weakness with clinically preserved sensation approximately 2 weeks after her last cycle of gemcitabine, oxaliplatin, leucovorin, and 5-fluorouracil (GOLF), rapidly progressing to an inability to ambulate.

**Diagnosis:**

Neurological examination and cerebrospinal fluid analysis (albuminocytologic dissociation) were consistent with GBS, and nerve conduction studies demonstrated absent motor and sensory responses, indicating a severe axonal variant (AMSAN); sensory involvement was electrodiagnostic rather than clinical. An extensive infectious, autoimmune, and paraneoplastic workup was negative, supporting a diagnosis of presumed oxaliplatin-related GBS; a Naranjo score of 4 indicated a “possible” drug association.

**Interventions:**

She received 5 sessions of therapeutic plasma exchange followed by 4 sessions of intravenous immunoglobulin.

**Outcomes:**

No meaningful neurological recovery occurred; she developed progressive respiratory failure requiring mechanical ventilation and died.

**Lessons:**

Clinicians should maintain a high index of suspicion for GBS in patients on platinum-based chemotherapy who develop abrupt, symmetric motor weakness after common causes have been ruled out. Because causality is difficult to establish, the association remains presumed; early recognition, prompt immunotherapy, and pharmacovigilance are essential to reduce mortality.

## 1. Introduction

Oxaliplatin is a third-generation diaminocyclohexane platinum analogue that exerts its antineoplastic effect through formation of platinum–DNA adducts and disruption of DNA synthesis. As a core component of combination chemotherapy regimens, it has contributed significantly to improved disease control and survival across advanced colorectal, hepatobiliary, and pancreatic malignancies.^[1,2]^ The National Comprehensive Cancer Network lists several oxaliplatin-based regimens as first-line therapies for metastatic colorectal cancer.^[1]^ Accordingly, more than half of patients with colorectal cancer receive oxaliplatin at some point in their disease course.^[1,2]^

Despite its efficacy, oxaliplatin is associated with well-characterized toxicities, most notably, nausea, vomiting, stomatitis, myelosuppression, and sensory neuropathy.^[3]^ This chemotherapy-induced neuropathy is further classified as acute or chronic. The acute form presents within hours to days of infusion as transient, cold-induced paresthesias, whereas the chronic form develops after cumulative exposure and manifests as persistent, symmetric sensory deficit.^[3,4]^ In both forms, motor involvement is uncommon, and progression is typically gradual rather than abrupt. Guillain-Barré Syndrome (GBS), by contrast, is an acute immune-mediated polyradiculoneuropathy characterized by progressive, symmetric weakness that may lead to respiratory failure and death if not promptly recognized and treated.^[5]^

Beyond its classic sensory neuropathy, oxaliplatin has been implicated in a small number of **cases of** GBS, including both demyelinating and axonal variants.^[6–9]^ We report a rare case of GBS in a patient receiving oxaliplatin as part of the gemcitabine, oxaliplatin, leucovorin, and 5-fluorouracil (GOLF) regimen for concurrent metastatic cholangiocarcinoma and colorectal carcinoma. Oxaliplatin-associated GBS is exceptionally uncommon, with only a handful of documented cases. Distinguishing this phenomenon from the far more common chemotherapy-induced neuropathy is critical for patient survival. We aim to highlight the importance of recognizing atypical features of neuropathy in patients undergoing platinum-based chemotherapy and stress the need for early consideration of immune-mediated etiologies when motor weakness develops.

## 2. Methodology

### 2.1. Ethics and consent

This single-patient case report was prepared in accordance with institutional policy. Institutional Review Board (IRB) approval was not required because this is a single, de-identified case report per institutional policy. Furthermore, the patient is deceased; despite reasonable efforts, informed consent could not be obtained from the next of kin. All information has been de-identified to protect patient privacy in accordance with applicable regulations.

### 2.2. Electrodiagnostic classification

For this case report, GBS subtype was classified using standard electrodiagnostic criteria based on nerve conduction studies (NCS). In particular, this methodology separates GBS into acute inflammatory demyelinating polyradiculoneuropathy (AIDP) with demyelinating features such as prolonged distal latencies, conduction slowing, and conduction block and axonal variants, such as acute motor axonal neuropathy (AMAN) and acute motor and sensory axonal neuropathy (AMSAN), characterized by reduced or absent compound muscle and sensory nerve action potential amplitudes.^[5]^

### 2.3. Literature review

We performed a structured search of PubMed on July 10, 2026, using the query (“Guillain-Barré syndrome” OR “Guillain-Barre syndrome”) AND (oxaliplatin OR cisplatin OR carboplatin OR platinum), which returned 22 records before deduplication (Fig. 1). After removal of one duplicate, 21 unique records were screened by title and abstract, and 14 were excluded (immune checkpoint inhibitor–associated GBS, n = 4; reviews, comparative studies, or trials, n = 4; paraneoplastic or non-drug-associated neuropathy, n = 2; correspondence or reply without a new case, n = 2; and unrelated topics, n = 2). The remaining 7 reports underwent full-text review and were included. The selection process is summarized in Figure 1. Six of the seven included reports contained extractable case-level data and are tabulated alongside the present case in Table 1 (references 6, 7, 14, 15, 16, and 17). The seventh included report (reference 8) is a brief report describing an axonal-form, oxaliplatin-associated case and is described in the Discussion. A subsequent correspondence item published in the same journal (reference 9), which comments on that report without describing an independent patient, was excluded and is accounted for within the “correspondence or reply without a new case” category in Figure 1.

**Table 1:** Literature review of chemotherapy-related Guillain–Barré syndrome cases.

Source	Age	Tumor	Platinum agent/ regimen	Onset → nadir (days)	CSF findings	ENMG pattern	Treatment	Outcome (follow-up)	Platinum rechallenge
Pappa, Berzero, Herlin et al–Patient #1	52	Ovarian adenocarcinoma	Carboplatin + paclitaxel	10	Normal	Sensory-motor axonal (23 d)	IVIG 2 g/kg	No motor deficit; mild distal paresthesias	Yes (carboplatin)
Pappa, Berzero, Herlin et al–Patient #2	62	Pancreatic adenocarcinoma	Oxaliplatin + 5-FU + folinic acid + irinotecan (FOLFIRINOX)	7	ACD (protein 130 mg/dL; cells 0/mm^3^)	Sensory axonal (7 d)	IVIG 2 g/kg	Complete recovery	No
Pappa, Berzero, Herlin et al–Patient #3	58	Pulmonary adenocarcinoma	Cisplatin + pemetrexed	3	Normal	Demyelinating (5 d)	None	No motor deficit; mild ataxia	Yes (carboplatin)
Pappa, Berzero, Herlin et al–Patient #4	58	Rectal adenocarcinoma	Oxaliplatin + 5-FU + capecitabine	11	ACD (protein 130 mg/dL; cells normal)	Demyelinating (17 d)	IVIG 2 g/kg	No motor deficit; mild ataxia	No
Pappa, Berzero, Herlin et al–Patient #5	69	SCC of penis	Cisplatin + 5-FU + docetaxel	8	ACD (protein 93 mg/dL; cells < 2/mm^3^)	Demyelinating (17 d)	IVIG 2 g/kg × 2	No motor deficit; intermittent paresthesias	No
Christodoulou et al	53	Colon adenocarcinoma	Oxaliplatin + 5-FU + leucovorin	14	ACD (protein 185 mg/dL; cells 2/mm^3^)	Demyelinating	IVIG 2 g/kg (×1)	1 wk later: no motor deficit; mild paresthesias	NR
Tho et al	63	Endometrioid endometrial carcinoma	Cisplatin	5	ACD (protein 110 mg/dL; cells normal)	Demyelinating	IVIG 2 g/kg (×2; 4-day interval)	1 mo: slight LL motor deficit (MRC 4/5)	NR
Cicero et al	55	NSCLC	Cisplatin + gemcitabine	7	ACD (protein 435 mg/dL; cells normal)	Demyelinating	IVIG 2 g/kg (×1)	Full recovery	Yes (carboplatin)
Verdonk et al	41	Nonseminomatous testis	Cisplatin + etoposide + bleomycin	7	ACD (protein 280 mg/dL; cells 0/mm^3^)	Demyelinating	IVIG 2 g/kg	Slow, gradual improvement	NR
Yoon et al	62	Cholangiocarcinoma	Oxaliplatin + 5-FU + leucovorin	14	ACD (protein 74.6 mg/dL; cells 1/mm^3^)	Demyelinating	IVIG 2 g/kg (×1)	2 mo: tetraparesis with paresthesias persists	NR
**Present case**	**77**	**Synchronous cholangiocarcinoma + colorectal adenocarcinoma**	**Oxaliplatin + gemcitabine + 5-FU + leucovorin (GOLF**)	**13**	**ACD (protein 80 mg/dL; cells 3/µL**)	**Severe axonal (absent responses**)	**PLEX × 5 then IVIG 2 g/kg × 4**	**Death**	**No**

5-FU = 5-fluorouracil; ACD = albuminocytologic dissociation (elevated protein with normal cell count); CSF = cerebrospinal fluid; ENMG = electroneuromyography; FOLFIRINOX = folinic acid, fluorouracil, irinotecan, oxaliplatin; GOLF = gemcitabine, oxaliplatin, leucovorin, 5-fluorouracil; IVIG = intravenous immunoglobulin; LL = lower limb; MRC = Medical Research Council (muscle strength grade); NR = not reported; NSCLC = non–small cell lung cancer; PLEX = plasma exchange; SCC = squamous cell carcinoma.

**Figure 1.**
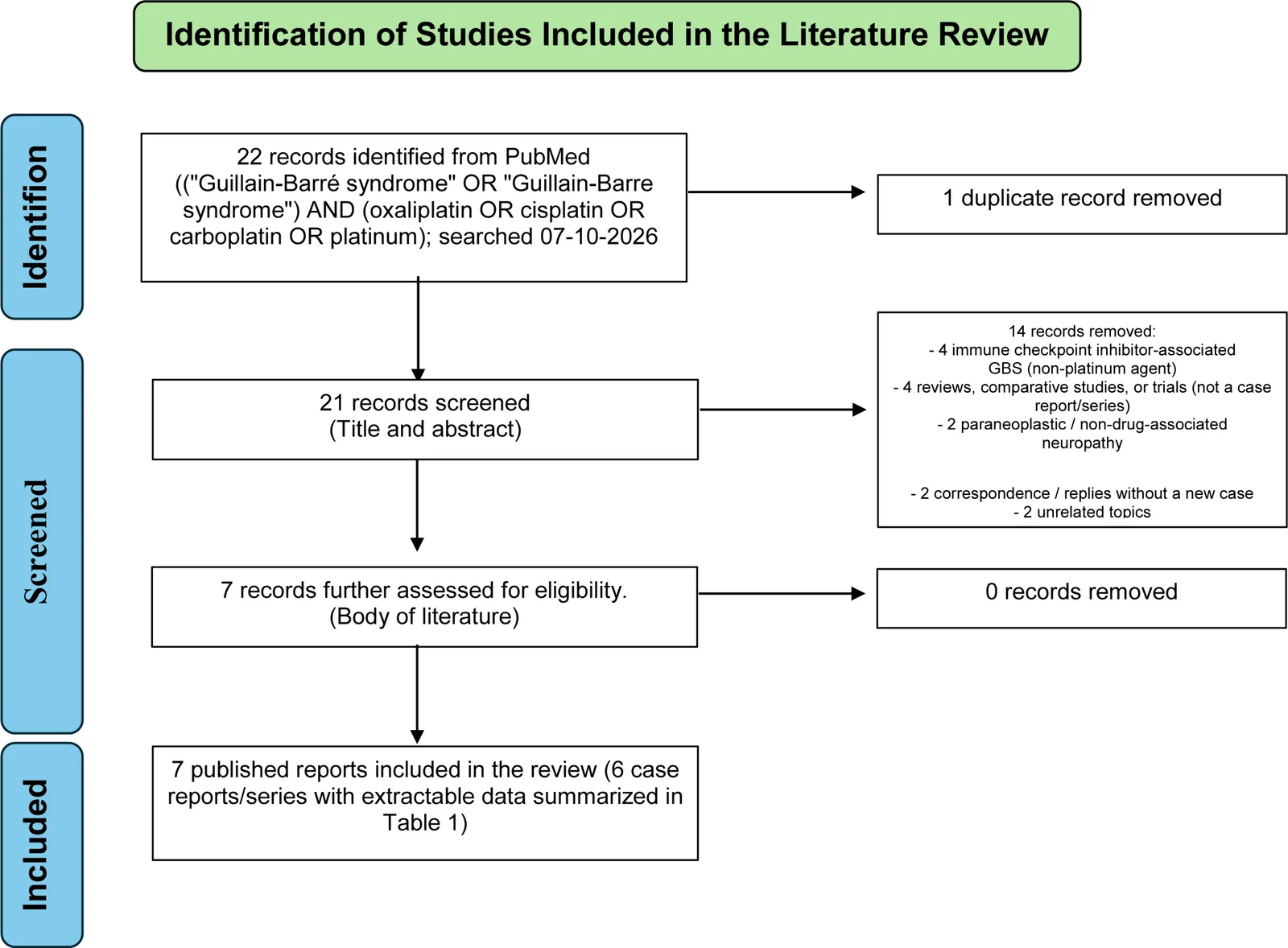
Flow diagram of study selection for the literature review of chemotherapy-related Guillain–Barré syndrome (PRISMA).

## 3. Case presentation

### 3.1. Admission and workup

A 77-year-old woman with a history of stage IV cholangiocarcinoma, stage IV colorectal cancer, hypothyroidism, and hyperlipidemia presented with acute-onset, generalized weakness. About 12 hours prior to admission, she abruptly developed symmetric weakness of her upper extremities, trunk, and lower extremities, resulting in an inability to ambulate and rapid progression to being bedbound. At baseline, she had been independently ambulatory and functionally active, prompting her family to seek immediate medical evaluation.

Her malignancies were diagnosed 6 months prior after evaluation for persistent abdominal pain. Imaging and subsequent biopsies revealed 2 synchronous primary malignancies: cholangiocarcinoma with hepatic invasion (T3NxM1) and colorectal carcinoma arising from the cecum with ovarian invasion (T2N1cM1a). At the time of diagnosis, her Eastern Cooperative Oncology Group (ECOG) performance status was one. She began a modified GOLF regimen administered every 2 weeks, with her most recent cycle administered 13 days prior to symptom onset. Home medications included antiemetics and opioids for cancer-related pain.

### 3.2. Investigation and diagnosis

On admission, the patient was fully alert and oriented. A neurological exam revealed diffuse, symmetric weakness (grade 3/5) across all muscle groups in both upper and lower extremities, with normal bulk but decreased tone. Gait was impaired with short, unsteady steps. Coordination was intact. Sensory examination, including light touch, pinprick, vibration, and proprioception, was preserved throughout and remained clinically intact for the duration of her admission. Deep tendon reflexes were 2 + in the upper extremities and diminished to 1 + at the patellae and ankles bilaterally, with downward plantar responses. She remained hemodynamically stable, and initial laboratory workup did not reveal any electrolyte or hematological derangements.

On day two of hospitalization, the patient experienced rapid functional deterioration with progression to complete flaccid paralysis (0/5 strength) in all extremities. Magnetic resonance imaging (MRI) of the brain, cervical, lumbar, and thoracic spine was unremarkable. An urgent lumbar puncture demonstrated clear cerebrospinal fluid with low nucleated cells, normal glucose, elevated protein, and negative cultures – consistent with albuminocytologic dissociation (Table 2). An extensive evaluation – including myositis antibodies, paraneoplastic panel, and autoimmune serologies – was unrevealing except for TSH of 26 IU/mL with a free T4 of 0.79 ng/dL (Table 3).

**Table 2:** Cerebrospinal fluid (CSF) results for lumbar puncture done during admission.

Parameter	Result	Reference Range
Appearance	Clear, colorless	Clear, colorless
Opening pressure	Not done	6–25 cm H_2_O
Red blood cells	3 cells/µL	0 cells/µL
Nucleated cells	3 cells/µL	0–5 cells/µL
Glucose	79 mg/dL	40–80 mg/dL
Protein	80 mg/dL	15–45 mg/dL
Gram stain (cytospin)	No organisms seen	No organisms detected
Culture	No growth	No growth observed

CSF glucose is interpreted relative to a simultaneous serum glucose; a CSF-to-serum glucose ratio of 0.6 or greater is considered normal. CSF = cerebrospinal fluid.

**Table 3:** Key laboratory results throughout the admission.

Test	Result	Reference Range
Vitamin B12	3300 pg/mL	200–900 pg/mL
Folate	25 ng/mL	≥3 ng/mL
Hemoglobin A1c	6.1 %	<5.7% = normal
Creatine kinase (CK)	19 U/L	26–140 U/L (adult female)
Thiamine (Vitamin B1)	137 nmol/L	70–180 nmol/L
TSH	26 µIU/mL	0.4–4.0 µIU/mL
Free T4	0.79 ng/dL	0.8–1.8 ng/dL
Aldolase	8.5 U/L	1.0–7.5 U/L
Vitamin D 25‑OH	50 ng/mL	30–100 ng/mL
Vitamin D 1,25‑OH	52.6 pg/mL	18–72 pg/mL
Campylobacter antibody	0.33	<1.10 = negative
West Nile virus serology	IgG negative; IgM negative	IgG = prior exposure; IgM = acute infection
Epstein–Barr virus serology	IgM negative; IgG positive	IgG = prior exposure; IgM = acute infection
Cytomegalovirus serology	IgM negative; IgG negative	IgG = prior exposure; IgM = acute infection
Influenza A/B PCR	Negative	-
HIV 1/2 Ag/Ab	Negative	-
ANA	Negative	-
ENA panel	Negative	-
Myositis panel (standard)	Negative	-
Extended myositis panel	Negative	-
Paraneoplastic antibody panel (Mayo)	Negative	-

Reference intervals are typical adult ranges. For viral serologies, a positive IgG with a negative IgM indicates prior exposure rather than acute or recent infection. Ag/Ab = antigen/antibody; ANA = antinuclear antibody; CMV = cytomegalovirus; EBV = Epstein–Barr virus; ENA = extractable nuclear antigen; Ig = immunoglobulin; PCR = polymerase chain reaction; VCA = viral capsid antigen.

### 3.3. Treatment and clinical course

By day four of hospitalization, the patient’s weakness progressed to diaphragmatic involvement, acute hypoxic respiratory failure, intubation, and transfer to the intensive care unit (ICU).

An early ABG on room air showed compensated but borderline gas exchange (pH 7.43, PaCO_2_ 43 mm Hg, PaO_2_ 78 mm Hg, HCO_3_^−^ 28 mEq/L, SaO_2_ 95%). As respiratory-muscle weakness progressed, she developed hypercapnic hypoventilation (pH 7.33, PaCO_2_ 55 mm Hg, PaO_2_ 68 mm Hg, HCO_3_^−^ 29 mEq/L) and then frank respiratory failure (pH 7.24, PaCO_2_ 68 mm Hg, PaO_2_ 58 mm Hg, HCO_3_^−^ 30 mEq/L). Concurrently, her negative inspiratory force (maximal inspiratory pressure) weakened to − 20 cm H_2_O, meeting accepted thresholds for neuromuscular respiratory failure and prompting intubation. Given the rapid progression of symmetric motor weakness, preserved sensory modalities, characteristic cerebrospinal fluid findings, and exclusion of infectious, metabolic, and paraneoplastic etiologies, a diagnosis of GBS was made. After multidisciplinary review, oxaliplatin was suspected as the precipitating agent.

At the time of maximal deficit, the patient was quadriplegic and ventilator-dependent, corresponding to a Hughes Disability Scale (HDS) grade of 5. The patient underwent 5 sessions of therapeutic plasma exchange, followed by 4 sessions of intravenous immunoglobulin, to treat her GBS. Despite this aggressive approach, no meaningful neurological improvement was observed. Nerve conduction studies, performed on hospital day 11 (approximately 11 days after symptom onset), after initiation of plasma exchange and prior to intravenous immunoglobulin, revealed severe axonal involvement with absent motor and sensory responses in all extremities, consistent with a severe axonal variant of GBS with AMSAN. Subsequent bedside examinations suggested sensory impairment; however, interpretation was limited by ongoing sedation and the patient’s inability to reliably participate while mechanically ventilated. After 2 weeks without neurological recovery, and in the context of persistent respiratory dependence and advanced malignancy, the patient’s family elected to pursue inpatient hospice care. The patient passed away shortly thereafter. The complete diagnostic and clinical course, from diagnosis, hospitalization, and outcome, is summarized in Figure 2.

**Figure 2.**
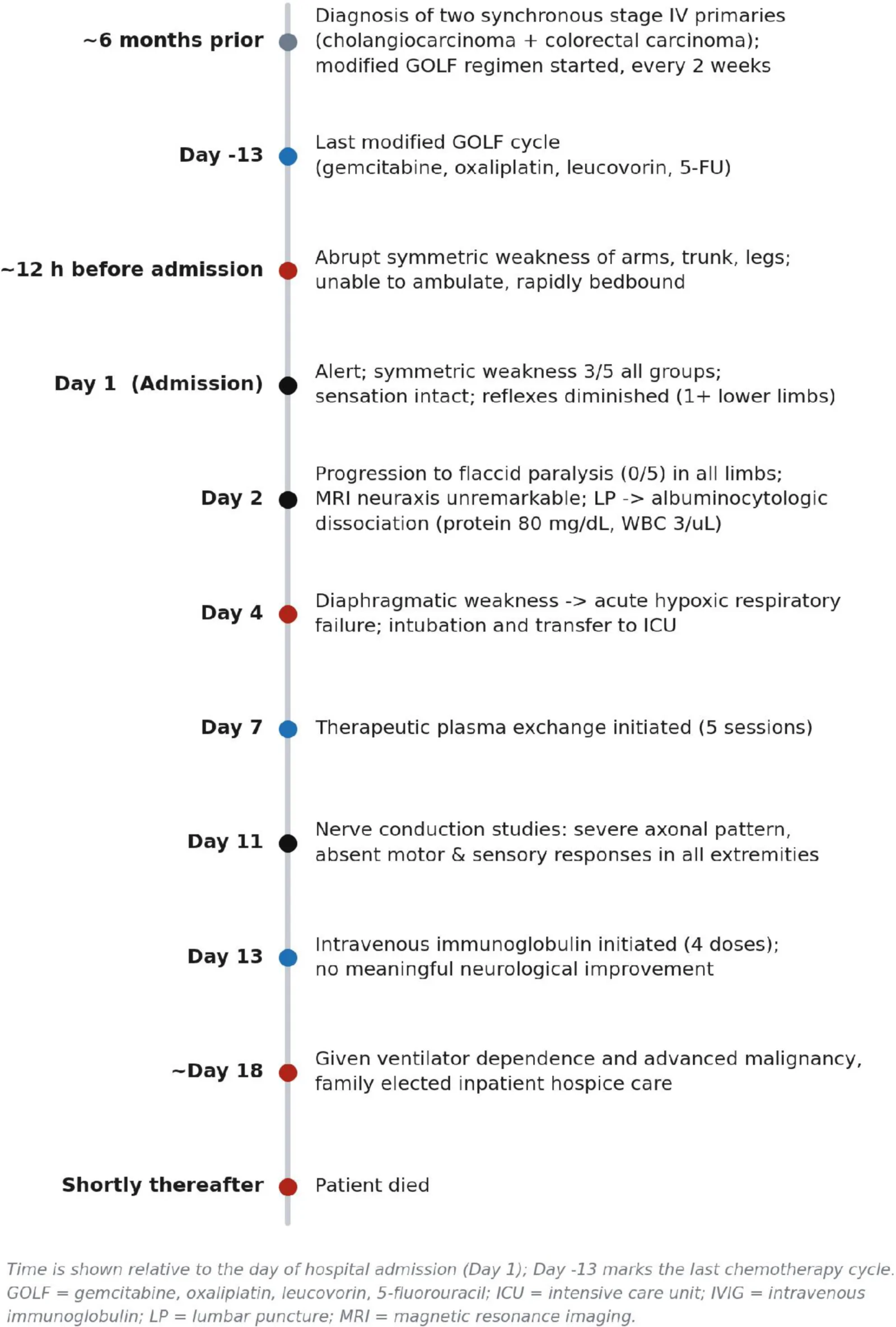
Diagnostic and clinical timeline of the patient’s course with presumed oxaliplatin-related Guillain–Barré syndrome.

## 4. Discussion

### 4.1. Guillain-Barré syndrome

We report a unique case of presumed oxaliplatin-related GBS. This acute polyneuropathy develops following the formation of immunoglobulin G (IgG) autoantibodies against gangliosides in myelinated axons of the peripheral nervous system; subsequent demyelination and decreased impulse transmission lead to motor and sensory dysfunction.^[5]^ It remains the most common cause of acute flaccid paralysis in the United States, with an annual incidence of ~1–2 per 100,000, and a broad clinical spectrum that includes classic acute inflammatory demyelinating polyradiculoneuropathy (AIDP) and motor or sensory-specific axonal variants.^[5,10]^

GBS often develops following infection, most commonly Campylobacter jejuni (C. jejuni) or viral illnesses such as cytomegalovirus (CMV), Epstein–Barr virus (EBV), Human immunodeficiency virus (HIV), and influenza.^[11]^ Certain vaccinations have also been associated with GBS, most notably the 1976 influenza vaccine.^[12]^ Diagnosis is largely clinical, characterized by rapidly progressive and symmetric weakness extending from the extremities to the trunk. Cerebrospinal fluid analysis and nerve conduction studies, while often done, are not required for diagnosis. When suspected, immediate treatment is needed to prevent respiratory failure and life-threatening autonomic dysfunction. Both IVIG and PLEX are equally effective treatments if administered within days of symptom onset.^[10]^ Our patient received both modalities sequentially without recovery, a result consistent with evidence that sequential therapy offers no additional benefit once a full course of either has been given.^[10]^ Prognosis is variable: approximately 25% of patients require mechanical ventilation, 4% to 15% die, and 20% have persistent disability.^[5,13]^

### 4.2. Drug-associated Guillain-Barré syndrome

Drug-associated GBS has been described with chemotherapies, immune checkpoint inhibitors, and fluoroquinolones. Among chemotherapies, platinum-based agents (oxaliplatin, cisplatin, and carboplatin) are commonly implicated.^[6,7]^ The largest published case series, by Pappa et al details 5 patients who developed GBS after platinum-based therapy for solid tumors (Table 1).^[6]^ Two received oxaliplatin-focused regimens, while three received unique cisplatin or carboplatin combinations.^[6]^ Symptoms developed at a median of 4 days after the last dose (compared to 13 days in our patient).^[6]^ Lumbar puncture showed albuminocytologic dissociation in three of 5 patients, and electromyography studies demonstrated demyelinating patterns in three of 5 patients.^[6]^ Most of the patients improved with discontinuation of the platinum agent and IVIG or PLEX, and two tolerated carboplatin rechallenge without recurrent neurotoxicity.^[6]^

In addition to this large case series, there are 5 individual case reports on chemotherapy-associated GBS (Table 1).^[7,14–17]^ In all cases, cerebrospinal fluid analysis demonstrated albuminocytologic dissociation, and electromyography revealed demyelinating features.^[7,14–17]^ Outcomes were generally favorable with platinum cessation and IVIG/PLEX; 1 patient tolerated carboplatin reintroduction well.^[15]^ The seventh included report, by Tyberghein et al., is of particular relevance to the present case, describing an axonal-form GBS arising during oxaliplatin-based chemotherapy.^[8]^ In contrast to the predominantly demyelinating cases summarized in Table 1, that report and the present case indicate that oxaliplatin-associated GBS may also manifest as a severe axonal phenotype with a less favorable prognosis. A subsequent correspondence item published in the same journal commented on that report without contributing an independent patient.^[9]^

### 4.3. Oxaliplatin neuropathy and mechanisms

Nearly 90% of patients who receive oxaliplatin will develop some degree of sensory neuropathy.^[4]^ Despite its efficacy, oxaliplatin is associated with well-characterized toxicities, most notably, nausea, vomiting, stomatitis, myelosuppression, and sensory neuropathy.^[3,18]^ This chemotherapy-induced neuropathy is further classified as acute or chronic. In the acute form, which occurs in 30% of patients per cycle, there is development of reversible, cold-triggered paresthesias and dysesthesias of the extremities.^[18]^ It usually manifests within hours of infusion and subsides over 10–14 days.^[18]^ In the chronic form, often lasting >14 days, there is cumulative accumulation of the oxaliplatin in the body that leads to persistent, symmetric sensory deficits.^[3,4]^

Diagnosing drug-associated GBS is challenging and requires strict exclusion of common precipitants, including infectious, inflammatory, and paraneoplastic phenomena. In our patient, testing for C. jejuni, HIV, EBV, CMV, and influenza was unrevealing. Furthermore, an extensive autoimmune, inflammatory, and paraneoplastic evaluation was unrevealing. Although thyroid dysfunction was present, the abrupt onset and rapid progression of symptoms were not consistent with hypothyroid-related neuropathy. Medication review identified no alternative temporally plausible triggers aside from oxaliplatin.

Taken together, the findings are compatible with a possible non-dose-dependent, immune-mediated mechanism rather than a directly neurotoxic etiology. One theory on this proposed mechanism details that platinum compounds, once infused, may form protein adducts (haptens) that are processed by antigen-presenting cells and presented to T-cells, driving downstream antiganglioside antibody production in susceptible hosts.^[19]^ A complementary hypothesis is molecular mimicry, whereby oxaliplatin-modified self-epitopes resemble gangliosides on myelinated fibers, precipitating an antibody-mediated attack identical to GBS.^[20]^

### 4.4. Alternative explanations and the role of malignancy

Causality in this case is presumptive, and alternative or contributing explanations warrant consideration. GBS has a higher reported incidence and worse prognosis in patients with active malignancy.^[21–23]^ In a nationwide case-control study, incident cancer was associated with a subsequently increased risk of GBS.^[21]^ A population-based cohort study reported that patients diagnosed with cancer within 1 year of GBS had markedly lower six-month survival than those without GBS (45% vs. 61%) and a higher adjusted mortality rate ratio (1.56; 95% CI, 1.27–1.90).^[22]^ A single-center study similarly found malignancy to be overrepresented among patients with GBS (odds ratio 2.08; 95% CI, 1.06–3.71) and associated with greater axonal loss and poorer neurological recovery.^[23]^ Hodgkin lymphoma, lung cancer, and colorectal cancer are among the most frequently implicated malignancies.^[21–23]^ Proposed mechanisms include paraneoplastic immune dysregulation and reduced immune tolerance in advanced or cachectic states, facilitating cross-reactive antibodies against peripheral-nerve gangliosides.^[22–24]^ In our patient, the presence of two synchronous advanced malignancies raises the possibility that a malignancy-related, immune-dysregulated state contributed to, or independently precipitated, GBS, an alternative that cannot be excluded and that tempers attribution to oxaliplatin alone.

### 4.5. Causality assessment

To formally assess the likelihood that oxaliplatin precipitated this event, we applied the Naranjo Adverse Drug Reaction Probability Scale.^[24]^ The temporal relationship between oxaliplatin exposure and symptom onset, the exclusion of alternative infectious and metabolic causes, and the biological plausibility of platinum-associated GBS support a causal contribution; however, the absence of neurological improvement after drug withdrawal, the lack of rechallenge, and the confounding presence of advanced malignancy limit certainty. The resulting Naranjo score of 4 corresponds to a “possible” adverse drug reaction, underscoring that oxaliplatin is a plausible but not definitively proven cause.

### 4.6. Prognostic considerations and limitations

Compared with the literature, in which most patients improved after IVIG and withdrawal of the suspected agent, our patient deteriorated despite treatment. Several adverse prognostic factors likely contributed. Her nerve conduction studies indicated a severe axonal pattern with absent motor and sensory responses, and axonal forms of GBS are generally associated with worse recovery than demyelinating (AIDP) forms. She was likely recognized and treated relatively late, with PLEX and IVIG initiated after diaphragmatic involvement and respiratory failure had developed; once respiratory failure occurs, mortality rises substantially, approaching 20% among ventilated patients.^[13]^ Finally, she had multiple synchronous primary malignancies, a condition itself associated with poor outcomes.^[25]^ The dissociation between preserved bedside sensory examination and absent sensory nerve action potentials in our patient is physiologically explicable. Routine sensory conduction studies assess only large-diameter myelinated (Aβ) fibers and are substantially more sensitive than clinical testing, such that considerable large-fiber loss may precede any detectable bedside deficit. Pain and temperature sensation are conveyed by small Aδ and C fibers, which are not assessed by routine nerve conduction studies. Clinical sensory findings may therefore lag behind or understate electrodiagnostic involvement, a pattern well recognized in AMSAN, in which the sensory component is frequently electrophysiologic rather than symptomatic. In our patient, this dissociation is further explained by the interval between the pre-intubation examinations and electrodiagnostic testing on hospital day 11, during which axonal degeneration progressed and formal sensory assessment was constrained by sedation and mechanical ventilation.

We acknowledge important limitations. This is a single case, and causation by oxaliplatin cannot be proven; the temporal association, exclusion of alternatives, and biological plausibility support but do not confirm attribution, as reflected by a Naranjo score in the “possible” range. Antiganglioside antibodies were not available to corroborate the proposed mechanism, and the coexistence of advanced malignancy is a potential confounder. Sensory examination was reliably assessable only during the early hospital course; following intubation on hospital day 4, formal sensory testing was limited by sedation and mechanical ventilation. The preserved sensory findings reported here therefore reflect the pre-intubation period, whereas electrodiagnostic testing was performed on hospital day 11. The interval between these assessments likely reflects both progression of axonal degeneration and a period during which bedside sensory testing could not be reliably performed. Electrodiagnostic studies were not repeated, and serial testing would have permitted more definitive subtype classification.

With the rising incidence of cholangiocarcinoma and colorectal cancer and the increasing use of platinum-based chemotherapy across inpatient and outpatient settings, oncologists and hospitalists must recognize that not all neuropathy in patients on oxaliplatin is sensory in nature. The abrupt onset of symmetric motor weakness represents a neurological emergency and should trigger evaluation for GBS. In such cases, clinicians should withhold the suspected agent, obtain urgent neurology consultation, initiate IVIG or plasma exchange without delay, and closely monitor respiratory and autonomic function. Because causality remains difficult to establish in individual cases, pharmacovigilance reporting and case aggregation are needed to define the incidence, mechanisms, and outcomes of this phenomenon. Future work should focus on multicenter registries with standardized data, observational cohorts to identify risk factors and early signals, and consensus guidance on the safety of resuming chemotherapy in affected patients.

## Author contributions

**Conceptualization:** Sikander Chohan, Muhammad Wasif Saif.

**Formal analysis:** Sikander Chohan, Muhammad Wasif Saif.

**Investigation:** Sikander Chohan, Muhammad Wasif Saif.

**Writing—original draft:** Sikander Chohan.

**Writing—review & editing:** Sikander Chohan, Muhammad Wasif Saif.

**Methodology:** Muhammad Wasif Saif.

**Supervision:** Muhammad Wasif Saif.

## References

[1] Stein A Arnold D. Oxaliplatin: a review of approved uses. Expert Opin Pharmacother. 2012;13:125–37. 10.1517/14656566.2012.643870 22149372

[2] Caraglia M Correale P Giannicola R. GOLFIG chemo-immunotherapy in metastatic colorectal cancer patients. a critical review on a long-lasting follow-up. Front Oncol. 2019;9:1102. 10.3389/fonc.2019.01102 PMC6857002 31781481

[3] Cavaletti G Marmiroli P. Management of oxaliplatin-induced peripheral sensory neuropathy. Cancers (Basel). 2020;12:1370. 10.3390/cancers12061370 PMC7352541 32471028

[4] Selvy M Pereira B Kerckhove N. Long-term prevalence of sensory chemotherapy-induced peripheral neuropathy for 5 years after adjuvant FOLFOX chemotherapy to treat colorectal cancer: a multicenter cross-sectional study. J Clin Med. 2020;9:2400. 10.3390/jcm9082400 PMC7465246 32727095

[5] Shahrizaila N Lehmann HC Kuwabara S. Guillain-Barré syndrome. Lancet. 2021;397:1214–28. 10.1016/S0140-6736(21)00517-1 33647239

[6] Pappa E Berzero G Herlin B. Guillain-Barré syndrome during platinum-based chemotherapy: a case series and review of the literature. Oncologist. 2020;25:e194–7. 10.1634/theoncologist.2019-0255 PMC6964130 31615948

[7] Christodoulou C Anastasopoulos D Visvikis A. Guillain-Barré syndrome in a patient with metastatic colon cancer receiving oxaliplatin-based chemotherapy. Anticancer Drugs. 2004;15:997–9. 10.1097/00001813-200411000-00010 15514570

[8] Tyberghein M Milants C Bouquiaux O Wang F. Axonal form of Guillain-Barré syndrome in a patient receiving oxaliplatin-based chemotherapy. Neurol Sci. 2020;41:1611–3. 10.1007/s10072-019-04199-2 31897938

[9] Jia L Zhang H. Axonal form of Guillain-Barré syndrome in a patient receiving oxaliplatin-based chemotherapy. Neurol Sci. 2021;42:365–6. 10.1007/s10072-020-04560-w 32705491

[10] Leonhard SE Mandarakas MR Gondim FAA. Diagnosis and management of Guillain-Barré syndrome in ten steps. Nat Rev Neurol. 2019;15:671–83. 10.1038/s41582-019-0250-9 PMC6821638 31541214

[11] Finsterer J. Triggers of Guillain-Barré syndrome: campylobacter jejuni Predominates. Int J Mol Sci . 2022;23:14222. 10.3390/ijms232214222 PMC9696744 36430700

[12] Vellozzi C Iqbal S Broder K. Guillain-Barre syndrome, influenza, and influenza vaccination: the epidemiologic evidence. Clin Infect Dis. 2014;58:1149–55. 10.1093/cid/ciu005 24415636

[13] Fletcher DD Lawn ND Wolter TD Wijdicks EF. Long-term outcome in patients with Guillain-Barré syndrome requiring mechanical ventilation. Neurology. 2000;54:2311–5. 10.1212/wnl.54.12.2311 10881259

[14] Tho LM O’Leary CP Horrocks I Al-Ani A Reed NS. Guillain-Barre syndrome occurring after adjuvant chemo-radiotherapy for endometrial cancer. Gynecol Oncol. 2006;100:615–7. 10.1016/j.ygyno.2005.09.008 16242761

[15] Cicero G Fulfaro F Caraceni A. A case of Guillain-Barré syndrome in a patient with non small cell lung cancer treated with chemotherapy. J Chemother. 2006;18:325–7. 10.1179/joc.2006.18.3.325 17129846

[16] Verdonk RC Enting RH Janmaat M Schroder CP Sleijfer DT Gietema JA. Weakness and numbness after chemotherapy for metastatic non-seminoma testis: a new neurological complication. Acta Oncol. 2008;47:1596–8. 10.1080/02841860701877330 18607855

[17] Yoon JY Nam TS Kim MK. Acute inflammatory demyelinating polyradiculoneuropathy in a patient receiving oxaliplatin-based chemotherapy. Asia Pac J Clin Oncol. 2012;8:201–4. 10.1111/j.1743-7563.2011.01515.x 22524580

[18] Oxaliplatin injection, powder, lyophilized, for solution. DailyMed. National Library of Medicine. Updated May 26, 20171. Accessed July 10, 2026. https://dailymed.nlm.nih.gov/dailymed/drugInfo.cfm?setid=d6178bc6-9dae-413f-8f47-de75d576c5b8

[19] Schuppe HC Haas-Raida D Kulig J Bömer U Gleichmann E Kind P. T-cell-dependent popliteal lymph node reactions to platinum compounds in mice. Int Arch Allergy Immunol. 1992;97:308–14. 10.1159/000236138 1597351

[20] Laman JD Huizinga R Boons GJ Jacobs BC. Guillain-Barré syndrome: expanding the concept of molecular mimicry. Trends Immunol. 2022;43:296–308. 10.1016/j.it.2022.02.003 PMC9016725 35256276

[21] Levison LS Thomsen RW Sindrup SH Andersen H. Association between incident cancer and Guillain-Barré syndrome development: a nationwide case-control study. Neurology. 2022;98:e1555–61. 10.1212/WNL.0000000000200015 35236772

[22] Girma B Farkas DK Laugesen K. Cancer diagnosis and prognosis after Guillain-Barré syndrome: a population-based cohort study. Clin Epidemiol. 2022;14:871–8. 10.2147/CLEP.S369908 PMC9309322 35898330

[23] Hiew FL Rajabally YA. Malignancy in Guillain-Barré syndrome: a twelve-year single-center study. J Neurol Sci. 2017;375:275–8. 10.1016/j.jns.2017.02.024 28320147

[24] Murali M Suppes SL Feldman K Goldman JL. Utilization of the Naranjo scale to evaluate adverse drug reactions at a free-standing children’s hospital. PLoS One. 2021;16:e0245368. 10.1371/journal.pone.0245368 PMC7806130 33439905

[25] Sanli AN Tekcan Sanli DE Altundag MK Aydogan F. Prognostic factors affecting survival of patients with single primary breast cancer vs patients with multiple primary cancers in lifetime, one of which is breast cancer. Am Surg. 2024;90:2745–55. 10.1177/00031348241248695 38676386

